# Cell-intrinsic differences between human airway epithelial cells from children and adults

**DOI:** 10.1016/j.isci.2022.105409

**Published:** 2022-10-20

**Authors:** Elizabeth F. Maughan, Robert E. Hynds, Adam Pennycuick, Ersilia Nigro, Kate H.C. Gowers, Celine Denais, Sandra Gómez-López, Kyren A. Lazarus, Jessica C. Orr, David R. Pearce, Sarah E. Clarke, Dani Do Hyang Lee, Maximillian N.J. Woodall, Tereza Masonou, Katie-Marie Case, Vitor H. Teixeira, Benjamin E. Hartley, Richard J. Hewitt, Chadwan Al Yaghchi, Gurpreet S. Sandhu, Martin A. Birchall, Christopher O’Callaghan, Claire M. Smith, Paolo De Coppi, Colin R. Butler, Sam M. Janes

**Affiliations:** 1 Lungs for Living Research Centre, UCL Respiratory, University College London, London WC1E 6JF, UK; 2 Epithelial Cell Biology in ENT Research (EpiCENTR) Group, Developmental Biology and Cancer Department, UCL Great Ormond Street Institute of Child Health, University College London, London WC1N 1DZ, UK; 3 University College London Cancer Institute, University College London, London WC1E 6DD, UK; 4 Infection, Immunity and Inflammation Department, UCL Great Ormond Street Institute of Child Health, University College London, London WC1E 1EH, UK; 5 Tracheal Service, Great Ormond Street Hospital, London WC1N 3JH, UK; 6 The National Centre for Airway Reconstruction, Department of Otolaryngology, Charing Cross Hospital, London W6 8RF, UK; 7 University College London Ear Institute, University College London, London WC1X 8EE, UK; 8 Stem Cell and Regenerative Medicine Section, University College London Great Ormond Street Institute of Child Health, University College London, London WC1N 1DZ, UK

**Keywords:** Medical science, Pediatrics, Molecular biology

## Abstract

The airway epithelium is a protective barrier that is maintained by the self-renewal and differentiation of basal stem cells. Increasing age is a principle risk factor for chronic lung diseases, but few studies have explored age-related molecular or functional changes in the airway epithelium. We retrieved epithelial biopsies from histologically normal tracheobronchial sites from pediatric and adult donors and compared their cellular composition and gene expression profile (in laser capture-microdissected whole epithelium, fluorescence-activated cell-sorted basal cells, and basal cells in cell culture). Histologically, pediatric and adult tracheobronchial epithelium was similar in composition. We observed age-associated changes in RNA sequencing studies, including higher interferon-associated gene expression in pediatric epithelium. In cell culture, pediatric cells had higher colony formation ability, sustained *in vitro* growth, and outcompeted adult cells in a direct competitive proliferation assay. Our results demonstrate cell-intrinsic differences between airway epithelial cells from children and adults in both homeostatic and proliferative states.

## Highlights

• Human airway epithelium was similar in composition in children and adults
• Interferon-associated transcriptional profile in pediatric airway mucosa
• Pediatric cells expanded more readily than adult cells in primary cell culture

## Graphical abstract

**Figure:**
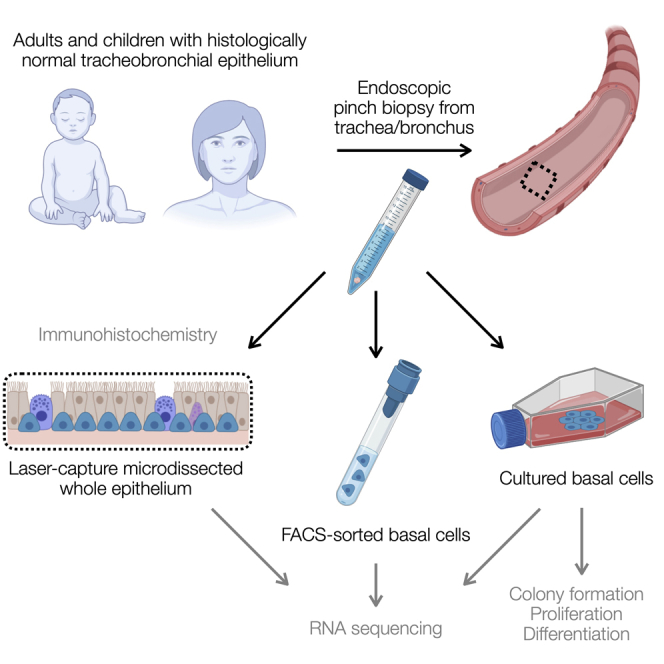

## Introduction

The proximal human airways are lined by a pseudostratified epithelium with diverse functions. The epithelium provides a structural barrier against inhaled particulates and pathogens, is a regulator of airway immune responses, and has the ability to replenish itself during cell turnover.^1^ These specialized functions are accomplished by luminal mucosecretory and ciliated epithelial cells, as well as basal stem cells, which act as multipotent progenitors.^2^^,^^3^ Although the structural and functional consequences of aging in the distal lung are fairly well characterized,^4^^,^^5^^,^^6^ little is known about age-associated alterations in human proximal airway epithelial cell composition or function. The importance of such age-associated changes has been highlighted by the COVID-19 pandemic, where functional differences in predisposition to viral infection and response have had a major clinical impact.

Single-cell RNA sequencing studies have shown that increased transcriptional noise and upregulation of a core group of age-associated molecular pathways—including protein processing- and inflammation-associated genes—are correlated with aging across mouse cell and tissue types, but additional processes are unique to particular cell types within specific organs, including the lungs.^7^^,^^8^ To date, however, such studies have not profiled the trachea, which has distinct composition and stem cell biology to the distal lung.^9^ In a study of murine tracheal aging, epithelial cell density was reduced and the proportion of basal cells within the epithelium was slightly decreased, but no obvious decline in basal cell *in vitro* clonogenic potential or differentiation capacity was observed.^10^ However, microarray gene expression analysis of bulk tracheal cells showed changes consistent with the development of low-grade chronic inflammation in the tracheas of older mice, together with an increased presence of activated adaptive immune cells.^10^

There are striking differences in airway structure and composition between rodents and humans,^11^ which suggests that the effects of aging on airway epithelial regeneration might differ substantially between species. Careful characterization of human pediatric and adult airway epithelial cell composition and function will inform both lung regenerative medicine efforts and our understanding of potential pathogenic mechanisms behind multiple chronic lung disease pathologies, as age is a risk factor for chronic obstructive pulmonary disease, pulmonary fibrosis, infection, and lung cancer.^12^^,^^13^^,^^14^ Airway basal stem cell dysfunction, perhaps accelerated by smoking, is likely to play a role in disease pathogenesis.^15^

Here, we compare homeostatic pediatric and adult human tracheobronchial epithelium in terms of cellular composition and gene expression profiles. We further investigate proliferative airway basal stem cell behavior in primary cell culture as a surrogate for behavior during regenerative responses.

## Results

### Homeostatic human tracheobronchial epithelium has comparable cellular composition in children and adults

**Figure 1:**
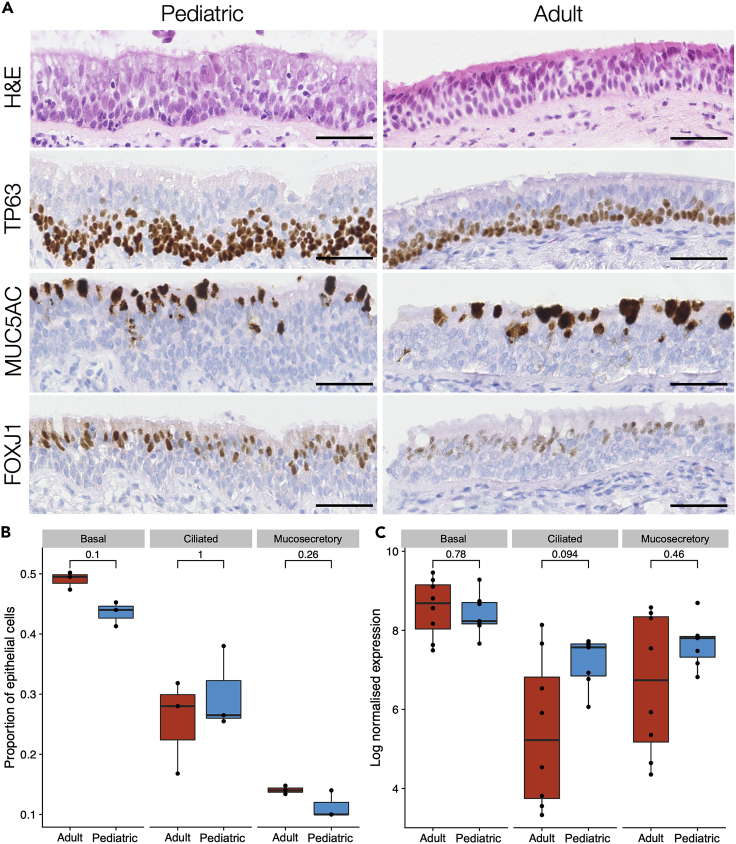
Cellular composition of pediatric and adult human tracheobronchial epithelium (A) Representative hematoxylin & eosin staining and immunohistochemistry comparison of TP63, MUC5AC, and FOXJ1 protein expression in pediatric and adult tracheobronchial epithelium. Scale bars = 50 μm. (B) Quantification of TP63^+^ basal cells, MUC5AC^+^ mucosecretory cells, and FOXJ1^+^ ciliated cells in pediatric and adult tracheobronchial epithelium from immunohistochemical data. Results are shown as a proportion of total cells within the epithelium (12,568 total cells for TP63, 9,651 for MUC5AC, and 16,144 for FOXJ1). No significant differences were seen in a two-sided Wilcoxon rank sum test (n = 3 donors/age group; basal cells, p = 0.1; ciliated cells, p = 1; mucosecretory cells, p = 0.26). (C) Expression of basal, ciliated, and mucosecretory cell markers in RNA sequencing data from laser capture-microdissected pediatric and adult epithelium. For each sample, the geometric mean of normalized counts of a set of cell type-specific gene markers (Table S2) is shown. No significant differences were seen in a two-sided Wilcoxon rank sum test (n = 7 pediatric and 8 adult donors; p = 0.78; ciliated cells p = 0.094, mucosecretory cells p = 0.46).

In mouse trachea, the proportion of the epithelium that expresses the basal cell-associated protein keratin 5 (KRT5) decreases with age,^10^ so we first compared the cellular composition of steady-state histologically normal human airway epithelium using hematoxylin and eosin (H&E) staining and immunohistochemistry for TP63 (basal cells), MUC5AC (mucosecretory cells), and FOXJ1 (ciliated cells) in tracheobronchial biopsies (Figures 1A and 1B; donor characteristics are listed in Table S1). During homeostasis, we found no significant differences in the proportion of cells in these three cellular compartments in pediatric and adult biopsies either by immunohistochemistry (Figures 1A and 1B), or by assessing basal, mucosecretory or ciliated cell-associated gene expression (Table S2) following bulk RNA sequencing of the laser capture-microdissected whole epithelium (Figures 1C and S1).

**Figure 2:**
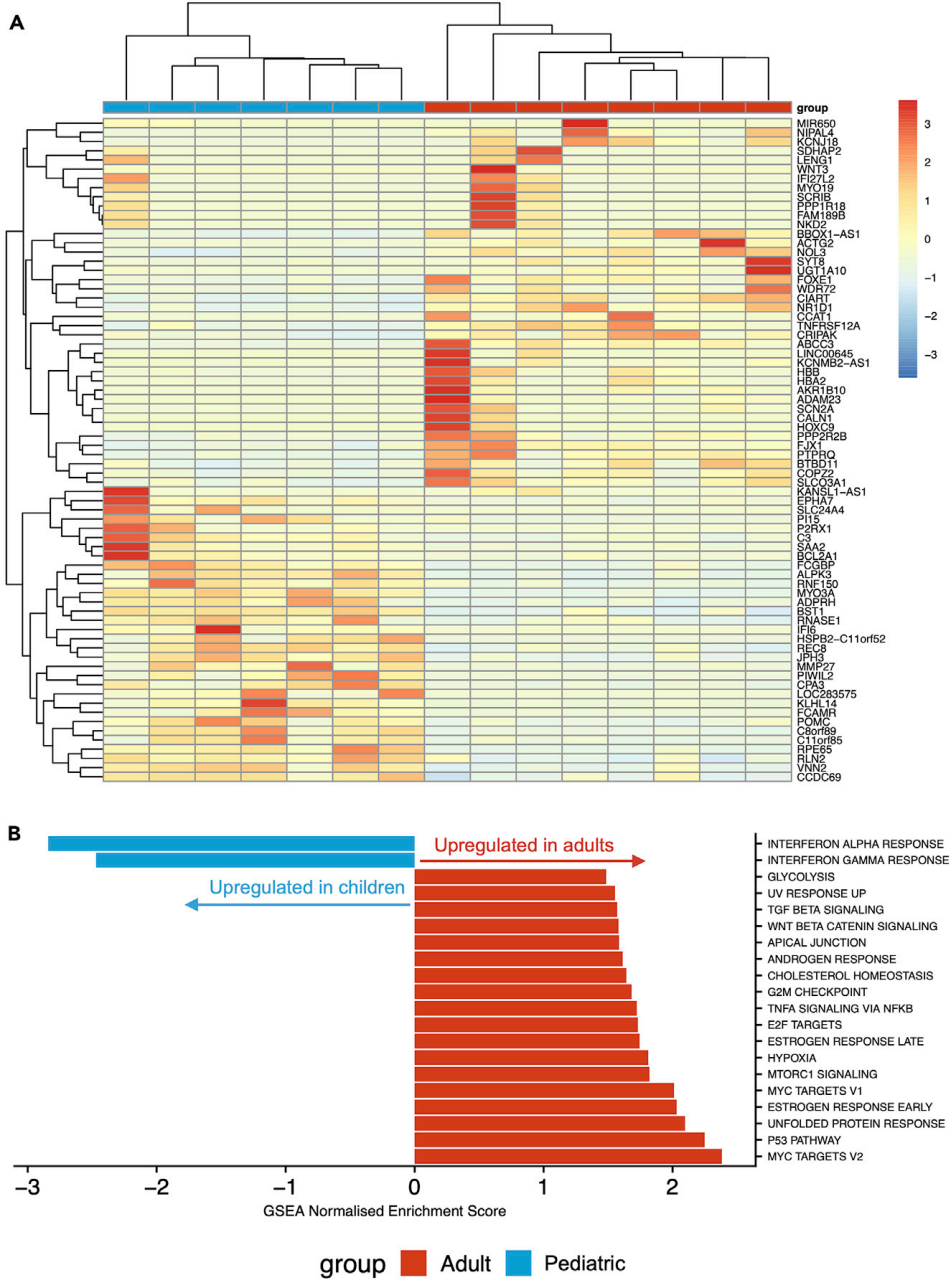
RNA sequencing of laser capture-microdissected whole epithelium from pediatric and adult proximal airways (A) Cluster diagram showing the normalized expression of all 72 genes differentially expressed with a false discovery rate (FDR) < 0.01 and a log_2_ fold change > 1.2 in seven pediatric (months of age/sex; 9F, 12M, 14M, 18M, 41M, 83M, and 106M) and eight adult (years of age/sex; 33F, 58F, 60F, 63M, 65M, 68F, 69M, and 72M) laser capture-microdissected tracheobronchial epithelial samples. Values are scaled by row. Gene order is based on hierarchical clustering based on the similarity in overall expression patterns. Red represents relative expression higher than the median expression and blue represents lower expression. (B) Pathway analysis was performed on the same pediatric and adult laser capture-microdissected tracheobronchial epithelial samples using gene set enrichment analysis (GSEA) to interrogate Hallmark pathways from MSigDB. For pathways with FDR <0.05, normalized enrichment scores are shown. A negative score (blue) represents upregulation of the pathway in the pediatric samples; a positive score (red) represents upregulation in the adult samples.

Analyzing this laser capture-microdissected whole epithelium RNA sequencing dataset using DESeq2^16^ with a false discovery rate (FDR) of 1% and log_2_ fold change threshold of 1.2, we identified 72 genes with significant differential expression between pediatric and adult donors, of which 32 were more highly expressed in children and 40 were more highly expressed in adults (Figure 2A and Table S3). To determine alterations in biologically functional gene groups, we performed gene set enrichment analysis (GSEA) using the Hallmark gene sets from MSigDB.^17^^,^^18^ This demonstrated a higher expression of genes associated with interferon alpha and gamma responses in the pediatric epithelium, potentially reflecting the pre-activated innate immune functions observed in pediatric airway epithelial cells *in vivo*.^19^ In adults, there was a higher expression of genes associated with TP53, mTORC1, Wnt-β-catenin, and TGFβ signaling, as well as processes such as cholesterol homeostasis and the unfolded protein response (Figure 2B).

**Figure 3:**
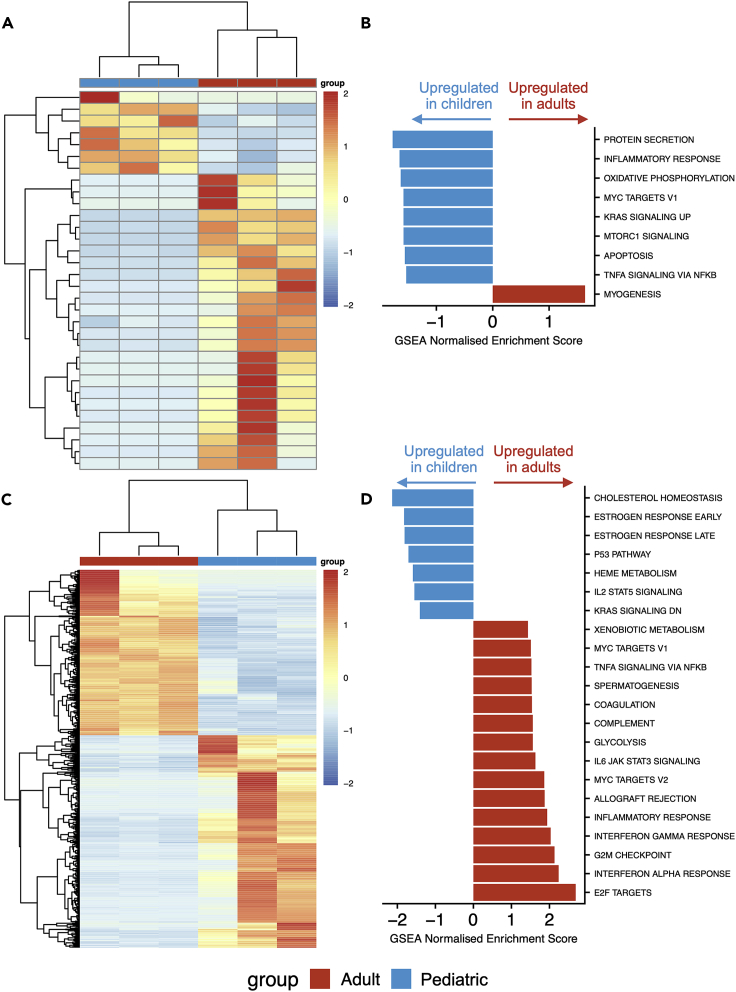
RNA sequencing of tracheobronchial basal cells from children and adults (A) Cluster diagram showing the expression of 32 differentially expressed genes with a false discovery rate (FDR) < 0.01 and a log_2_ fold change > 1.2 in three pediatric (months of age/sex; 2.5M, 11M, and 14M) and three adult (years of age/sex; 58F, 61F, and 62F) freshly fluorescence-activated cell-sorted EpCAM^+^/PDPN^+^ basal cell samples. Gene order is based on hierarchical clustering based on the similarity in overall expression patterns. Red represents relative expression higher than the median expression and blue represents lower expression. (B) Pathway analysis was performed on the three pediatric and three adult freshly fluorescence-activated cell-sorted EpCAM^+^/PDPN^+^ basal cell samples using gene set enrichment analysis (GSEA) to interrogate Hallmark pathways from MSigDB. For pathways with FDR < 0.05, normalized enrichment scores are shown. A negative score (blue) represents upregulation of the pathway in the pediatric samples; a positive score (red) represents upregulation in the adult samples. (C) Cluster diagram showing the expression of 987 differentially expressed genes with a false discovery rate (FDR) < 0.01 and a log_2_ fold change > 1.2 in three pediatric (months of age/sex; 3M, 30M, and 83M) and three adult (years of age/sex; 58F, 60F, and 69M) cultured basal cell samples. Cells were cultured on 3T3-J2 mouse embryonic fibroblast feeder layers for two or three passages in epithelial cell culture medium containing Y-27632. Gene order is based on hierarchical clustering based on the similarity in overall expression patterns. Red represents relative expression higher than the median expression and blue represents lower expression. (D) Pathway analysis was performed on the three pediatric and three adult cultured basal cell samples using gene set enrichment analysis (GSEA) to interrogate Hallmark pathways from MSigDB. For pathways with FDR < 0.05, normalized enrichment scores are shown. A negative score (blue) represents upregulation of the pathway in the pediatric samples; a positive score (red) represents upregulation in the adult samples.

Since basal cells act as stem cells in the proximal airways, we assessed whether there are gene expression differences between pediatric and adult basal cells by using a fluorescence-activated flow sorting (FACS) approach to isolate EpCAM^+^/PDPN^+^ basal cells^20^^,^^21^ directly from tracheal biopsies. Flow cytometry experiments in independent biopsies verified that the majority of keratin 5 (KRT5)-expressing basal cells were captured using this sorting strategy (Figure S2). Consistent with successful purification of basal cells, we saw clustering of sorted basal cells away from laser capture-microdissected whole epithelium (Figure S3A). Using DESeq2 with an FDR of 1% and log_2_ fold change threshold of 1.2, we identified 32 genes with significant differential expression between basal cells sorted from pediatric and adult donors, of which 7 were more highly expressed in children and 25 were more highly expressed in adults (Figure 3A and Table S3). The majority of differentially expressed genes do not have previously described roles in airway basal cells. However, *NTRK2*, which was more highly expressed in adult compared to pediatric basal cells, has previously been associated with basal cell function as it was upregulated in basal cells isolated from human nasal polyps compared with basal cells from the normal nasal epithelium.^22^ GSEA suggested that pathways such as TNFα and MTORC1 signaling, as well as processes such as inflammation and apoptosis, were higher in pediatric basal cells, although all pathways were of borderline statistical significance in this analysis (Figure 3B).

### Proliferating cultured basal cells demonstrate greater age-related transcriptional differences than basal cells *in vivo*

Given the relatively modest differences in gene expression seen in whole epithelium and FACS-sorted basal cells, we established primary cell cultures in which proliferation of airway basal cells was induced *in vitro* in order to assess whether additional differences between pediatric and adult basal cells manifest in regenerative conditions. We performed bulk RNA sequencing on cultured basal cells that were isolated and expanded on 3T3-J2 mouse embryonic feeder cells in epithelial cell culture medium containing Y-27632.^23^^,^^24^^,^^25^ Freshly sorted basal cells were more similar to laser capture-microdissected whole epithelium than cultured basal cells (Figure S3A), emphasizing the significant impact of the proliferation-inducing cell culture environment on the basal cell transcriptome. As expected, cultured cells were enriched for basal cell-associated genes compared to laser capture-microdissected whole epithelium (Figure S3B). Using DESeq2 with an FDR of 1% and log_2_ fold change threshold of 1.2, we identified 987 genes with significant differential expression between cultured basal cells from pediatric and adult donors, of which 555 were more highly expressed in children and 432 were more highly expressed in adults (Figure 3C and Table S3). GSEA suggested differences in multiple pathways between pediatric and adult cultured basal cells, some of which, including TNFα signaling and the inflammatory response, were now in the opposite direction to what had been seen in basal cells *in vivo* (Figure 3D).

**Figure 4:**
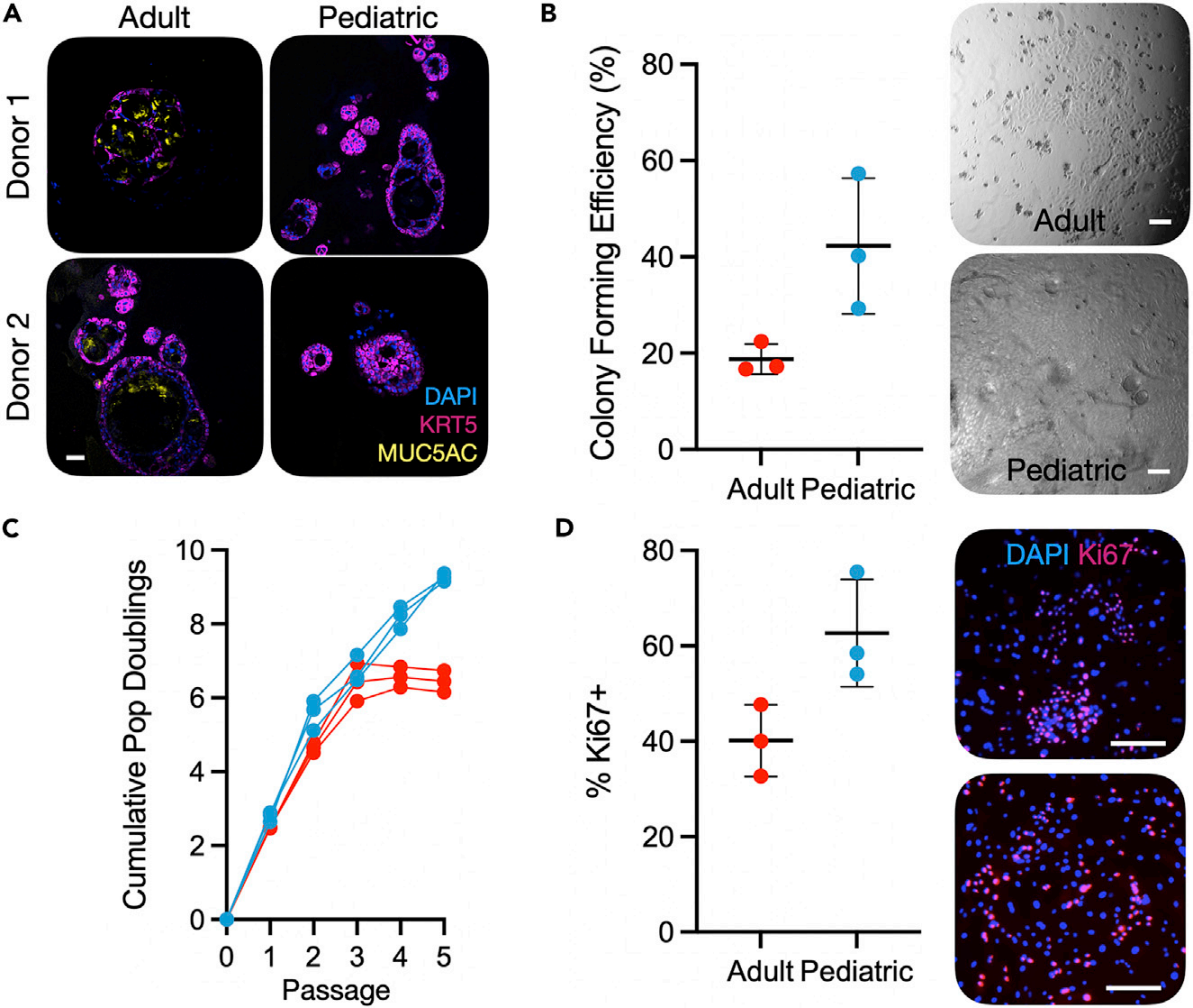
Altered proliferation and differentiation potential in pediatric airway basal cells compared to adult basal cells (A) Immunofluorescence images showing tracheosphere cultures derived from two pediatric and two adult basal cell cultures. Sections were stained with primary antibodies against keratin 5 (KRT5; purple), MUC5AC (yellow), and a nuclear counterstain (blue). Scale bar = 50 μm. (B) Proportion of EpCAM^+^/PDPN^+^ basal cells that formed colonies within 7 days after sorting single cells into 96-well plates in epithelial cell culture medium containing Y-27632 (mean ± SD; n = 3 donors/age group; n = 167–192 cells per donor; p = 0.048, two-tailed unpaired t-test). Brightfield microscopy images show a confluent pediatric well and an adult colony after 7 days of culture. Scale bar = 50 μm. (C) Population doubling analysis of the growth of pediatric and adult basal cell cultures in epithelial growth medium without Y-27632. Pediatric samples outperformed adults at passage 5 (p = 9.8 × 10^−5^, two-tailed unpaired t-test). (D) Ki67 immunofluorescence staining of passage one pediatric and adult basal cells cultured on feeder layers in epithelial cell culture medium without Y-27632 for 3 days before fixation. Ki67 positivity was quantified as a proportion of positive nuclei/total DAPI stained nuclei (mean ± SD; n = 3 donors/age group; mean of 1402 cells counted per donor, range 810 – 1600; p = 0.045, two-tailed unpaired t-test). The upper panel is an adult culture and the lower panel a pediatric culture.

Notably, the mucins *MUC2*, *MUC3A*, *MUC5AC*, *MUC5B*, and *MUC17*, as well as the secretory master regulator *SPDEF*, were all more highly expressed by adult cultured basal cells than by pediatric basal cells. We have previously observed upregulation of mucosecretory genes, such as *SCGB3A1*, in adult basal cells in these culture conditions compared to those in a serum-free alternative, bronchial epithelial growth medium.^23^ However, even if mucosecretory gene expression is favored in these conditions, it is unclear why pediatric and adult epithelial cells differ in their response to culture. We hypothesized that these age-associated effects might be more pronounced after the induction of differentiation. Consistent with this, we observed that adult basal cells produced more mucous in 3D tracheosphere cultures than pediatric cells (Figures 4A and S4A).

### Basal cells from children proliferate more readily in primary cell culture than those from adults

To investigate whether these transcriptional differences mirror functional differences between pediatric and adult basal cells, we fluorescence-activated cell-sorted single EpCAM^+^/PDPN^+^ basal cells, into individual wells of 96-well plates (range = 167 to 192 cells per donor) to compare the potential of freshly sorted pediatric and adult basal cells to generate colonies. Consistent with our previous work,^26^ after 7 days of culture in epithelial cell culture medium containing Y-27632, colony formation was significantly higher among basal cells derived from children than adults (Figure 4B). Indeed, at this timepoint, pediatric basal cells had often generated colonies that had become confluent to fill the well, whereas no adult colonies reached confluence (Figure 4B and S4B).

When cells were isolated and cultured in epithelial cell culture medium without Y-27632 on 3T3-J2 feeder layers, expansion of pediatric and adult cells proceeded similarly at early passages but a growth advantage was observed in pediatric donors after 5 passages (Figure 4C). Ki67-positivity in passage one cultures was higher in pediatric than adult cultures and orthogonal MTT- (Figure S4C) and EdU-based (Figure S4D) proliferation assays supported this finding. When cultured basal cells were assessed in colony formation assays, there was a trend toward pediatric cells forming more colonies than adult cells (Figure S4E).

### Basal cells from children outcompete those from adults in mixed cultures

**Figure 5:**
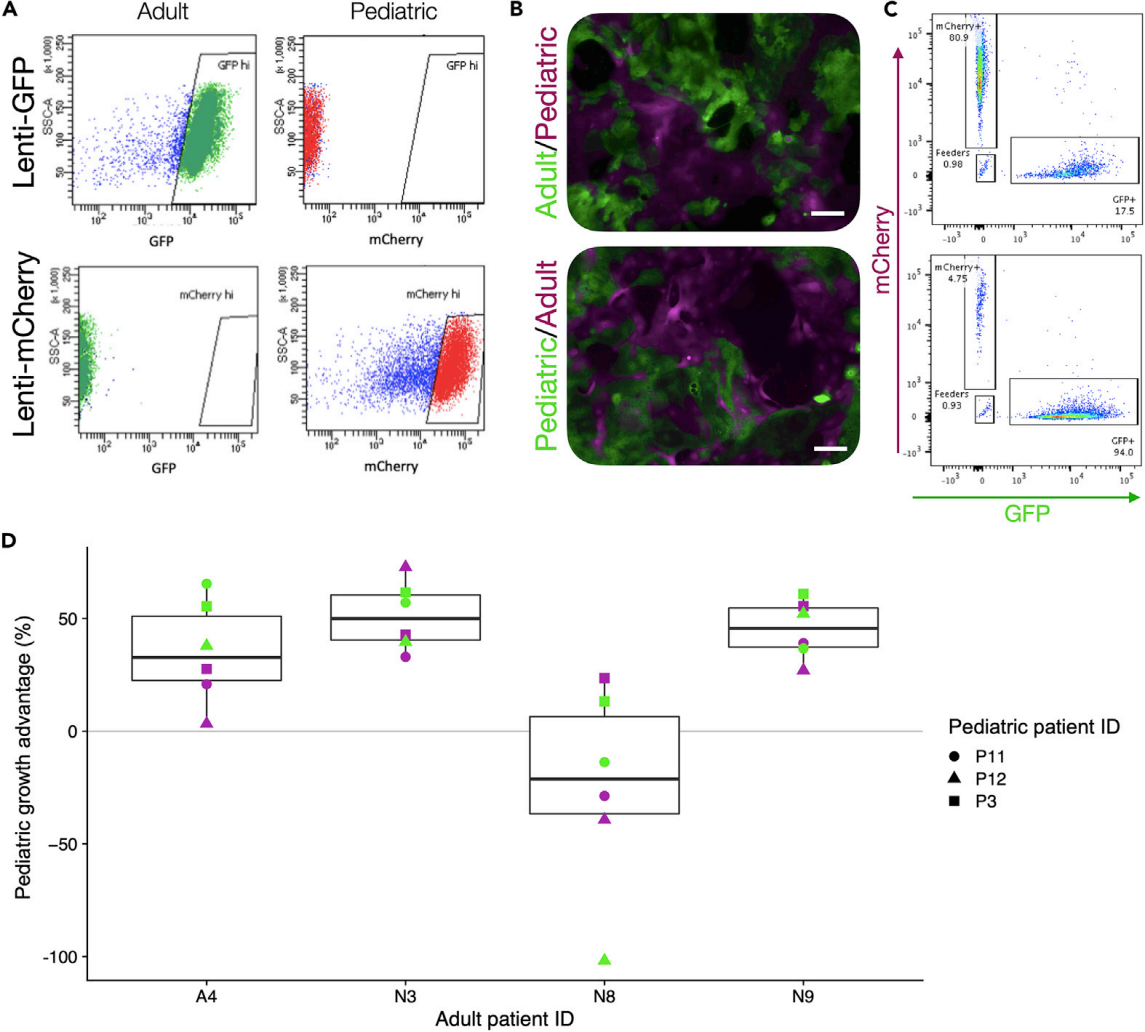
Pediatric airway basal cells outcompete adult basal cells in mixed cultures (A) GFP^+^ and mCherry^+^ proximal airway basal cells were generated from pediatric (n = 3) and adult (n = 4) donors by lentiviral transduction and purified by fluorescence-activated cell sorting (FACS). (B) Representative immunofluorescence demonstrates how each pediatric donor was mixed with each adult donor in a 1:1 ratio by FACS in 24-well plates containing 3T3-J2 feeder cells and epithelial cell culture medium containing Y-27632. Scale bars = 200 μm. (C) Flow cytometric analysis was performed when cultures reached approximately 80% confluence. As shown, this allowed quantification of the abundance of GFP^+^/mCherry^+^ and GFP^−^/mCherry^−^ feeder cells in these co-cultures. (D) Summary data for co-cultures between each pediatric and adult cell culture pair. The growth advantage of pediatric donors relative to adult donors is shown. Experiments were performed in technical triplicate and mean values for both GFP (pediatric)/mCherry (adult) and mCherry (pediatric)/GFP (adult) co-cultures are plotted as green circles and purple circles, respectively, to control for possible differential effects of the specific viruses used on cell proliferation. Donor sex and age are as follows: P3 = M, 6 years old; P11 = M, 2 years old; P12 = F, 1 year old; A4 = M, 63 years old; N3 = F, 58 years old; N8 = F, 30 years old; N9 = F, 62 years old.

To better understand the differences in progenitor capacity between pediatric and adult cultures, we next developed a competitive proliferation assay (Figure S5), using lentiviral cell labeling with fluorescent constructs.^27^ After optimization in 293T cells to ensure that the two lentiviruses did not affect cell growth (Figure S6), we isolated and cultured patient basal cells in epithelial cell culture medium containing Y-27632 to facilitate lentiviral transduction^28^ and transduced these with either green fluorescent protein (GFP)- or mCherry-expressing lentiviral constructs (Figure 5A). When combining GFP^+^ and mCherry^+^ cells from the same donor in equal number, the ratio remained 1:1 over 7 days (Figure S7D). After transducing three pediatric and four adult basal cell cultures in this manner, we combined each pediatric donor with each adult donor in both possible color combinations so that we could monitor the growth dynamics of the two populations separately using fluorescence (Figures 5B and 5C). There were no differences in lentiviral integration as determined by PCR targeting the puromycin resistance gene contained within both GFP and mCherry lentiviral vectors (Figure S7E). When cells were harvested at approximately 80% confluence, the growth differential between pediatric cells and adult cells was calculated for each pediatric/adult pair, considering any baseline differences between the cultures in monoculture. In almost all of the donor pairs, the pediatric cells outgrew the adult cells, although the pairings involving the youngest adult donor, who was 30 years of age, did not follow this pattern (Figure 5D).

## Discussion

In this study, we explored differences between human tracheobronchial basal cells in children and adults in three bulk transcriptomic experiments; we compared homeostatic laser capture-microdissected whole epithelium, homeostatic fluorescence-activated cell-sorted basal cells, and proliferating cultured basal cells. At the level of the whole epithelium during homeostasis, there was broad conservation of airway epithelial transcriptional programs. However, notable differences in the expression of genes associated with interferon responses were observed. While consistent with the existence of a primed innate immune response in pediatric airway epithelial cells that might be associated with enhanced antiviral response,^19^ we also saw a trend toward increased frequency of intraepithelial CD45^+^ cells in pediatric compared to adult epithelium (Figure S1F) that may also explain this finding.

Basal cell-specific RNA sequencing revealed few differences in gene expression between pediatric and adult tracheobronchial basal cells *in vivo* that might explain the differences in their proliferative capacity once cultured, although the role of many of the differentially expressed genes has not been determined in respiratory epithelial cells. Pathway analysis suggested that, consistent with bulk epithelium, inflammatory responses are higher in pediatric basal cells, suggesting that innate immune functions of basal cells might also be more active in children than adults. Upon culture, substantial transcriptomic changes were induced in airway basal cells, demonstrating one of the limitations of airway cell culture models.^29^ However, our finding that culture conditions may affect basal cells from children differently to those from adults raises questions about whether cells from children are better able to buffer the effects of the artificial environment than those from adults. As an example, inflammatory response pathways were upregulated in adult basal cells compared to pediatric basal cells, although it has previously been shown that pediatric basal cells in these culture conditions can mount an inflammatory response upon stimulation with poly I:C.^30^ These age-specific changes induced by culture should be a consideration in disease modeling using primary cells, particularly as donor age also affected the production of the mucin MUC5AC in the 3D tracheosphere model.

Functional differences were observed in epithelial cell cultures where basal cells from children had a greater colony-forming capacity and proliferation than those from adults. Moreover, they had a competitive advantage in mixed cultures that was greater than would be expected from their proliferation rates as single cultures. These data are consistent with studies in other epithelia, where aging reduces the proportion of cells identified as stem cells using *in vitro* methodologies.^31^ Identifying whether these findings reflect *in vivo* loss of progenitor capacity with aging will be important, since this might be responsible for the differing repair responses following airway injury between children and adults.^32^ The molecular mechanisms by which airway basal cells lose their *in vitro* proliferative capacity with age also warrant attention, particularly given our conflicting observations that pathways associated with the inflammatory response, MYC signaling, and TNF alpha signaling are more highly expressed in pediatric than adult basal cells *in vivo* but more highly expressed in adult than pediatric basal cell cultures, potentially revealing a limitation of the current *in vitro* system. These data are also of relevance for lung regenerative medicine. For example, pediatric cells might be more amenable to engraftment following transplantation, as is the case in bone marrow transplantation, where donor age significantly affects outcomes.^33^ Although future airway epithelial cell therapies are likely to be predominantly required by older people and be autologous in nature, recognizing age-related differences in regenerative capacity might allow the development of approaches that improve the culture and transplantation of aged basal cells. Furthermore, corrective gene and cell therapies in the context of genetic diseases such as cystic fibrosis^34^ might be more efficient if performed early in life, when cultured cells have greater progenitor potential.

Overall, our results demonstrate cell-intrinsic differences between proximal airway epithelial cells of children and adults in both homeostatic and proliferative states. These age-specific differences, particularly those seen in culture, argue for donor age to be a consideration where primary airway cells are used in disease modeling.

### Limitations of the study

Laser capture-microdissection has been used as a tool to analyze gene expression within the airway epithelium by multiple groups^35^^,^^36^^,^^37^^,^^38^ but this approach will have captured intraepithelial immune cell populations in addition to epithelium. While we do not see significant changes in the overall number of CD45^+^ cells within the epithelium between children and adults (Figure S1F), there was a trend toward CD45^+^ being more abundant in pediatric epithelium which may affect our interpretation of the RNA sequencing experiment presented in Figure 2. Additionally, age-associated composition or gene expression changes within these intraepithelial CD45^+^ cells may also contribute to the observed increased abundance of immune-related transcripts.

The small size of the endobronchial biopsies obtained in our study limited the extent to which we could perform multiple analyses per patient and resulted in some analyses having a relatively low number of pediatric or adult samples. It would be desirable to validate our findings in a larger cohort and assess the extent to which different cell culture conditions (2D culture vs. organoids; media compositions) reveal differences between pediatric and adult cultures.

## STAR★Methods

### Key resources table

**Table:** 

REAGENT or RESOURCE	SOURCE	IDENTIFIER
**Antibodies**		

Anti-CD31 (BV421)	Biolegend	303124; RRID:AB_2563810
Anti-CD45 (BV421)	Biolegend	304031; RRID:AB_10900423
Anti-PDPN (PE-Cy7)	Biolegend	337013; RRID:AB_2563367
Anti-KRT5 (AF-488)	Abcam	193894; RRID:AB_2893023
Anti-Ki67	Thermo Fisher Scientific	RM-9106; RRID:AB_2341197
Anti-MUC5AC	Sigma-Aldrich	M5293; RRID:AB_2146985
Anti-TP63	Abcam	ab735; RRID:AB_305870
Anti-FOXJ1	eBioscience	14-9965-80

**Biological samples**		

Human tracheobronchial blocks	This study	

**Chemicals, peptides, and recombinant proteins**		

CellFIX	BD Biosciences	340181
Permeabilization Buffer	Life Technologies	C10419
jetPEI	Polyplus Transfection	101–01N
PEG-it Virus Precipitation Solution	System Biosciences	LV810A-1
Zombie Violet Fixable Viability Kit	Biolegend	423113
Y-27632	Cambridge Bioscience	Y1000
Adenine	Sigma-Aldrich	A2786
Hydrocortisone	Sigma-Aldrich	H0888
EGF	Sino Biological	10605
Insulin	Sigma-Aldrich	I6634
Cholera Toxin	Sigma-Aldrich	C8052
T3	Sigma-Aldrich	T5516
Rat Tail Collagen I	BD Biosciences	354236

**Critical commercial assays**		

PureLink HiPure Plasmid Maxiprep Kit	Thermo Fisher Scientific	K210006
Click-iT EdU Staining Kit (Alexa Fluor 488)	Life Technologies	

**Deposited data**		

RNA sequencing data	GEO	GEO: GSE148818

**Experimental models: Cell lines**		

Human: primary tracheobronchial epithelial cell cultures	This study	N/A
Human: 293T cells	Available from ATCC	CRL-3216
Mouse: 3T3-J2 mouse embryonic fibroblasts	Fiona Watt (King’s College London)	

**Oligonucleotides**		

Puromycin Copy Number (Custom Taqman Probes)	Taqman	4331348

**Recombinant DNA**		

Plasmid: pCDH-EF1-copGFP-T2A-Puro	Addgene	#72263
Plasmid: pCDH-CMV-mCherry-T2A-Puro	Addgene	#72264
Plasmid: pMDLg/pRRE	Addgene	#12251
Plasmid: pRSV-Rev	Addgene	#12253
Plasmid: pMD2.G	Addgene	#12259

### Resource availability

#### Lead contact

Further information and requests for resources and reagents should be directed to and will be fulfilled by the lead contact, Prof. Sam M. Janes (s.janes@ucl.ac.uk).

#### Materials availability

Primary human cell cultures derived in this study are available to academic researchers via material transfer agreement as per the terms of the ethical approval under which they were established.

#### Data and code availability

RNA sequencing datasets associated with study have been deposited at GEO and are publicly available as of the date of publication (GEO: GSE148818). The code associated with data analyses in this manuscript are available via GitHub (https://github.com/ucl-respiratory/maughan_2022).

### Experimental model and subject details

#### Patient samples

Ethical approval to obtain patient tracheobronchial biopsies was granted by the National Research Ethics Committee (REC references 11/LO/1522 and 06/Q0505/12) and patients (or their parents/guardians) gave informed, written consent. Luminal biopsies of clinically normal tracheobronchial mucosa were obtained using cupped biopsy forceps from patients undergoing planned rigid laryngotracheobronchoscopy under general anaesthesia or flexible bronchoscopy under sedation. Patient characteristics, procedure indication and precise site of biopsy are included in Table S1.

#### Human airway epithelial cell culture

Primary human airway epithelial cells were isolated and expanded on mitotically inactivated 3T3-J2 feeder layers in two previously reported epithelial growth media, one containing Y-27632^23^^,^^39^ and one without.^40^^,^^41^ Feeder layers were prepared as previously described.^24^ Epithelial cell culture medium without Y-27632 consisted of Dulbecco’s modified Eagle’s medium (DMEM)/F12 in a 3:1 ratio containing 1× penicillin–streptomycin, 10% fetal bovine serum, 1% adenine, hydrocortisone (0.5 μg/mL), EGF (10 ng/mL), insulin (5 μg/mL), 0.1 nM cholera toxin, 2 × 10^−5^ T3 and gentamicin (10 mg/mL; Gibco), as previously described.^41^ Epithelial cell culture medium containing Y-27632 consisted of DMEM/F12 in a 3:1 ratio containing 1× penicillin–streptomycin (Gibco), 5% fetal bovine serum (Gibco) supplemented with 5 μM Y-27632, hydrocortisone (25 ng/mL), epidermal growth factor (0.125 ng/mL), insulin (5 μg/mL), 0.1 nM cholera toxin, amphotericin B (250 ng/mL; Thermo Fisher Scientific) and gentamicin (10 μg/mL; Gibco), as previously described.^23^ Where indicated, dishes were collagen I-coated by diluting rat tail collagen I (BD Biosciences) to 50 μg/mL in sterile 0.02N acetic acid and applying at 5 μg/cm^2^ for one hour at room temperature in a tissue culture hood. Coated surfaces were washed once with sterile PBS before cell seeding. Population doublings were calculated as previously described.^23^

### Method details

#### Histology

Samples for histology were fixed overnight in 4% paraformaldehyde (PFA) in PBS before being dehydrated through an ethanol gradient using a Leica TP 1050 vacuum tissue processor. Samples were embedded in paraffin and sectioned at 5 μm thickness using a microtome. Hematoxylin and eosin (H&E) staining was performed using an automated system (Tissue-Tek DRS, Sakura). Immunohistochemistry was performed by The Queen Mary University of London Barts Cancer Institute Pathology Service using the Ventana DabMap Horseradish Peroxidase Kit. Stained slides were scanned using a Nanozoomer Whole Slide Imager (Hamamatsu Photonics) to create virtual slides using NDP.View2 software. For cell type quantification, images of sections that contained areas of intact epithelium with at least 300 cells were used (overall 1–6 slides were assessed per donor). Images were reviewed in Fiji software and positively stained cells counted using the cell count function. In total, 12,568 (TP63), 9,651 (MUC5AC), 16,144 (FOXJ1) and 17,459 (CD45) cells were assessed for expression of these proteins.

#### Bulk RNA sequencing of tracheobronchial epithelium

Bronchoscopic biopsies were frozen immediately in optimal cutting temperature compound (OCT; in liquid hexane or on dry ice) and transported to a histopathology laboratory (Great Ormond Street Children’s Hospital, London, U.K.) within 2 h on dry ice. Blocks and cut slides were stored at −80°C prior to use. 10 μm sections were mounted on MembraneSlide 1.0 PEN (D) membrane covered slides (Zeiss). One H&E slide was cut per block to aid navigation and identification of epithelium and basement membrane. Slides were prepared with serial washes in methanol, RNAse-free water, RNAse inhibitor and ethanol to remove residual OCT. Laser capture microdissection was performed using a PALM MicroBeam 4 Laser Microdissection microscope at 10× and 20× magnification (Figure S1) to extract the epithelial portion (or all cells above the basement membrane) from each biopsy into microadhesive-capped tubes (Zeiss). Samples were suspended in a 2:1 mix of Arcturus PicoPure Extraction buffer: RNAlater (Life Technologies) and stored at −80°C until use.

For RNA extraction, samples were thawed, disrupted by lysis (incubation at 42°C for 30 min followed by incubation at room temperature for 5 min), vortexed and filtered using RNAeasy MinElute columns (Qiagen). RNA extraction was performed using the Arcturus PicoPure RNA Isolation Kit (Life Technologies Ltd; KIT0204) as per the manufacturer’s instructions. RNA was quantified using the Qubit RNA HS Assay Kit (Thermo Fisher Scientific). Libraries were created by UCL Genomics Core Facility using the SMARTer Stranded Total RNAseq Kit (Clontech), cleaned using JetSeq (Bioline) and quality control analysis of RNA integrity was performed using High Sensitivity RNA ScreenTape and the TapeStation Analysis software (Agilent Technologies). RNA sequencing was performed using 0.5× NextSeq for 75 cycles (Illumina; 43PE, ∼33M reads per sample). RNA sequencing QC analysis is shown in Table S4.

#### Fluorescence-activated cell sorting of basal cells

Samples were transported to the laboratory in transport medium consisting of αMEM containing penicillin/streptomycin, amphotericin B and gentamicin. Cell suspensions were generated by sequential enzymatic digestion using dispase 16 U/mL (Corning) for 20 min at room temperature followed by 0.1% trypsin/EDTA (Sigma) for 30 min at 37°C (both in RPMI medium, Gibco). Each enzyme step was quenched with medium containing FBS, placed on ice and combined following the second digestion. Biopsies were manually homogenized using sharp dissection between digest steps and by blunt homogenization through a 100 μm cell strainer (Miltenyi Biotec). Centrifugation steps were performed at 300 × g for 5 min at 4°C. Cells were blocked in a fluorescence-activated cell sorting (FACS) buffer composed of PBS containing 1% FBS, 25 mM HEPES buffer and 1 mM EDTA for 20 min in 96-well V-bottomed plates (Thermo Fisher Scientific). Cells were centrifuged as above before staining for 20 min on ice at 4°C. For colony formation assays and bulk RNA sequencing, the antibodies used were CD31 (BV421; Biolegend; 303124), CD45 (BV421; Biolegend; 304031), EpCAM (APC; Biolegend; 324208), PDPN (PE-Cy7; Biolegend; 337013). For flow cytometry experiments verifying that sorted populations were KRT5+ basal cells, cells were treated as above then fixed using CellFIX (BD Biosciences; 340181), permeabilized with a saponin-based reagent (Life Technologies, C10419) before intracellular staining with an anti-KRT5 antibody (AF-488; Abcam; 193894). Cells were resuspended in FACS buffer for sorting.

For colony formation assays, basal cells were sorted into collagen I-coated 96-well plates containing 3T3-J2 feeder cells at 20,000 cells per cm^2^ using a BD FACSAria Fusion FACS sorter running BD FACSDiva 8.0 software at the UCL Cancer Institute Flow Cytometry Core Facility. Experiments lasted 7 days and at termination, the number of wells that had become confluent was counted manually using a light microscope. Brightfield images were taken using a Zeiss Axiovert A1 microscope.

For bulk RNA sequencing, basal cells were sorted into epithelial cell culture medium containing Y-27632 for transport to the laboratory before being centrifuged at 300 × g for 5 min at 4°C and resuspended in RNA extraction buffer and processed as above for laser capture-microdissected samples.

#### RNA sequencing of cultured basal cells

RNA sequencing of cultured basal cells was performed after either two or three passages in epithelial cell culture medium containing Y-27632. RNA was extracted using the RNeasy Mini Kit (Qiagen) following the manufacturer’s instructions. RNA was submitted for library preparation by the Wellcome-MRC Cambridge Stem Cell Institute. Initial QC was performed using Qubit RNA HS Assay Kit (Thermo Fisher Scientific) and TapeStation Analysis software (Agilent Technologies). 600 ng RNA was used for library preparation using the NEBNext Ultra8482 II Directional RNA Library Prep Kit (Illumina) and the QIAseq FastSelect RNA Removal Kit (Qiagen). Libraries were subsequently measured using the Qubit system and visualized on a TapeStation D5000. Sequencing was performed using the NovaSeq6000 SP PE50 Standard (∼58M Illumina reads per sample) at the CRUK-CI Genomics Core. Following quality control and adapter trimming with fastp (as described above), paired-end reads were aligned to the human genome UCSC hg38 using RNA-STAR^42^ version 2.5.2b. Count matrices were obtained using featureCounts. Downstream analysis was performed as described for epithelial and basal RNAseq datasets above.

#### RNA sequencing analysis

Following sequencing, run data were demultiplexed and converted to FASTQ files using Illumina’s bcl2fastq Conversion Software v2.19. Quality control and adapter trimming were performed using fastp^43^ version 0.20.1 with default settings. FASTQ files were then tagged with the UMI read (UMITools^44^) and aligned to the human genome UCSC hg38 using RNA-STAR^42^ version 2.5.2b. Aligned reads were UMI deduplicated using Je-suite^45^ version 1.2.1 and count matrices were obtained using featureCounts. Downstream analysis was performed using the R statistical environment version 3.5.0 with Bioconductor version 3.8.0.^46^ Batch effects between two groups of samples run separately were identified and corrected using the *ComBat* method within the *sva* Bioconductor package.^47^ Counts were compared between pediatric and adult groups using DESeq2^16^ using the default settings. Where normalized counts were required, default DESeq2 normalization was applied. Pathways were assessed using an implementation of gene set enrichment analysis (GSEA) in the fgsea R package,^48^ using Hallmark gene sets from MSigDB^17^^,^^18^ as input. Heatmaps were plotted using the pheatmap^49^ package, implementing a complete linkage clustering method. All other plots were created using ggplot2. To avoid confounding by sex distribution, differences between the pediatric and adult groups, genes on the X and Y chromosomes were removed prior to differential analysis.

#### Identification of gene lists

This study utilizes several gene lists as included in Table S2: markers of basal, secretory and ciliated cells, viral response genes and COVID-19 genes of interest. Viral genes were identified following expert review of the literature. For cell marker genes we followed the method of Danaher and colleagues^50^ to identify consistently expressed markers across age groups, based on the assumption that genes consistently associated with a cell type should correlate with each other. Candidate markers for each cell type were derived from a large single cell dataset,^51^ by taking all genes significantly over-expressed (with FDR <0.01) in clusters associated with the given cell type. These candidate genes were then tested against healthy lung RNAseq data from the LungMAP dataset (downloaded from https://lungmap.net/breath-omics-experiment-page/?experimentTypeId=LMXT0000000018&experimentId=LMEX0000003691&analysisId=LMAN0000000342&view=allEntities; accessed on June 3, 2020), representing samples from infants, children and adults. For each list of candidate genes, we constructed a similarity matrix in each age-specific dataset following the Danaher method and performed hierarchical clustering. The final set of marker genes for each cell type were defined as those present in the main co-correlating cluster of all three age-specific datasets.

#### Proliferation assays

For MTT assays, primary human airway epithelial cells that had been isolated and expanded in epithelial cell culture medium without Y-27632 were seeded in 96-well plates at a density of 5,000 cells/well for 24 h without feeder cells. At the stated end-points, adherent cells were stained with MTT dye solution (10 μL of 1:10 diluted MTT stock solution in culture medium) for 3 h at 37°C. After incubation, the medium was removed and 100 μL dimethyl sulfoxide (DMSO; Sigma-Aldrich) was added to dissolve the MTT crystals. The eluted specific stain was measured using a spectrophotometer (560 nm).

To analyze EdU uptake, passage 1 primary human airway epithelial cells that had been isolated and expanded in epithelial cell culture medium without Y-27632 were cultured until approximately 70% confluence. Cells were washed with PBS and feeder cells were removed by differential trypsinization. After washing in DMEM containing 10% FBS and then PBS again, the remaining epithelial cells were treated with 10 μM EdU (Life Technologies Click-iT EdU Alexa Fluor 488) for 1 h. Cells were trypsinized to obtain single cell suspensions, stained according to the manufacturer’s instructions and finally co-stained with DAPI. Cells were run on an LSRFortessa (BD Biosciences) flow cytometer and data were analyzed using FlowJo 10.0.6 (TreeStar).

#### Colony formation assays

2,000 cultured human airway epithelial cells were seeded per well of a six-well plate containing inactivated 3T3-J2 feeder cells. Medium was carefully changed on day 4 and day 8 of culture before the experiment was terminated on day 12. Colonies were fixed for 10 min in 4% PFA in PBS, stained using crystal violet (Sigma-Aldrich) at room temperature for 20 min and washed repeatedly in water. Colonies of more than 10 cells were counted manually using a light microscope. Colony forming efficiency was calculated as: (number of colonies formed/number of seeded cells) ∗ 100.

#### Immunofluorescence

Cells were cultured in 8-well chamber slides (Ibidi) and fixed in 4% PFA in PBS for 20 min at room temperature. Slides were washed and stored in PBS until staining. Cells were permeabilized and blocked in PBS containing 10% FBS and 0.025% Triton X- for 1 h at room temperature. Primary anti-Ki67 antibody (Thermo Fisher Scientific, RM-9106) was incubated overnight in block buffer without Triton X- at 4°C. After three 5-min washes in PBS, anti-rabbit secondary antibodies (AlexaFluor dyes; Molecular Probes) were incubated at a 1:200 dilution in block buffer without Triton X- for 2 h at room temperature. Cells were washed in PBS, counter-stained using DAPI (1 μg/mL stock, 1:10,000 in PBS) and washed twice more in PBS. Images were acquired using a Zeiss LSM700 confocal microscope.

Ki67 staining was performed by seeding passage 1 cells on feeder cells for 3 days. Feeder cells were removed by differential trypsinization and basal cells were fixed using 4% PFA in PBS prior to staining as above. Ki67 positivity was assessed by manual counting of Ki67-stained nuclei as a proportion of all DAPI-stained nuclei in five images per donor (mean = 1402 cells per donor, range 810–1600).

#### GFP and mCherry lentiviral production

One Shot Stbl3 chemically competent *E*. *coli* bacteria (Thermo Fisher Scientific) were transformed using third generation lentiviral plasmids. The GFP-containing and mCherry-containing plasmids were pCDH-EF1-copGFP-T2A-Puro and pCDH-CMV-mCherry-T2A-Puro (gifts from Kazuhiro Oka; Addgene plasmids #72263 and #72264). The packaging plasmids used were pMDLg/pRRE, pRSV-Rev and pMD2.G (gifts from Didier Trono; Addgene plasmids #12251, #12253 and #12259^52^). Bacteria were plated on ampicillin-containing agar plates overnight, and colonies expanded from this in LB broth containing ampicillin. Plasmids were extracted using the PureLink HiPure Plasmid Maxiprep Kit (Thermo Fisher Scientific) with the PureLink HiPure Precipitator module (Thermo Fisher Scientific) as per the manufacturer’s instructions. Plasmid DNA concentration was quantified using a NanoDrop system.

293T Human Embryonic Kidney (HEK) cells grown in DMEM containing 10% FBS and 1× penicillin/streptomycin were used to assemble and produce the viruses. Packaging and transfer plasmids were delivered into 293T cells by transfection with jetPEI (Polyplus; 101–01N) as per the manufacturer’s instructions. Medium was changed at 4 and 24 h post-transfection. Culture medium containing the shed virus was collected at 72 h and incubated with PEG-it virus precipitation solution (System Biosciences) at 4°C for 48 h. The precipitated virus was pelleted by centrifugation of the mixture at 4°C and resuspended as per the manufacturer’s instructions.

Viral stock titre was determined by incubating 293T HEK cells (50,000 cells/well) in 12-well plates at 1:100, 1:1000, 1:10,000 and 1:100,000 viral dilutions in complete DMEM medium containing 4 μg/mL polybrene for 4 h before medium was refreshed. After 72 h, cells were trypsinized, DAPI stained and GFP or mCherry positivity was determined by flow cytometry. Viral titre was calculated as:

Viral titre (particles/mL) = [(% positive cells) x 50,000]/Volume of virus added (mL)

#### Competitive growth assay optimization in 293T HEK cells

293T HEK cells were transduced for 24 h with either mCherry (multiplicity of infection (MOIs) of 0.15 or 0.75) or GFP viruses (MOIs of 0.05 or 0.5). Following incubation for 72 h, cells were sorted by FACS into high- or low-expressing populations and expanded further. Population doubling times were calculated for each color and MOI, and these values were compared to untransduced cells. Cells were then seeded as a 1:1 mix of GFP- and mCherry-expressing cells into quadruplicate wells of a 48-well plate (10,000 of each color at either high or low expression). These cells were passaged twice per week. At each passage, the contents of each well were trypsinized and the percentage of GFP and mCherry-expressing cells was analyzed by flow cytometry. 80% of each well’s cell volume was immediately re-plated into fresh 48-well plates whilst 20% of the sample from each well was stained with a live/dead fixable stain, fixed with 4% PFA in PBS and analyzed for GFP and mCherry expression by flow cytometry. Reference wells containing 10,000 cells of a single color were trypsinized at the same time points and counted manually to calculate a doubling time for each cell type.

#### Lentiviral transduction of cultured human basal cells

Primary human tracheobronchial basal cells were isolated and expanded in epithelial cell culture medium containing Y-27632 for two passages before transduction with either GFP- or mCherry-containing viruses (MOI = 100). Transduction was performed in culture medium plus 4 μg/mL polybrene and medium was exchanged for fresh culture medium after 16 h. Purification of cultures to remove untransduced cells was performed by FACS for GFP or mCherry positivity after 7–10 days of further expansion. Since both the GFP and mCherry lentiviral plasmids carry a puromycin resistance cassette, PCR copy number analysis for the puromycin cassette was performed to quantify the number of lentiviral copies incorporated per donor cell (Taqman custom copy number assay). Genomic DNA (gDNA) was extracted from each transduced cell culture using a blood & cell culture DNA mini kit (Qiagen) as per the manufacturer’s instructions. gDNA was quantified using a NanoDrop system and PCR performed using the TaqMan genotyping master mix and reference assay (Life Technologies). Stable transduction was confirmed by flow cytometry prior to competitive proliferation assays.

#### Human airway basal cell competitive proliferation assays

Feeder cells were removed from cultures of GFP^+^ and mCherry^+^ basal cells by differential trypsinization and basal cells were suspended in FACS buffer containing 5 μM Y-27632 for FACS. Cells were seeded by FACS as a 1:1 mix of GFP- and mCherry-expressing cells into triplicate wells of a 24-well plate containing 3T3-J2 feeder cells (5000 or 10,000 of each color). All pediatric cultures were crossed with all of the adult cultures of the opposite label. Medium was refreshed after three and five days. At seven days or at 80–90% confluence as ascertained by fluorescence microscopy, the entire contents of the well were trypsinized for flow cytometry (i.e. feeder cells were not removed). The resulting cell suspensions were digested enzymatically with 0.25 U/mL liberase (thermolysin medium; Roche) in serum-free medium to form a single cell suspension, which was quenched with medium containing 10% FBS. Zombie Violet Live/Dead fixable stain was applied as per the manufacturer’s instructions and cell suspensions were fixed with 4% PFA in PBS for 15 min at room temperature. GFP and mCherry expression was assessed by flow cytometry. Feeder cells were seen as an unlabeled population. Untransduced pediatric cells cultured in parallel were used as non-labelled controls and single-color wells containing 10,000 cells from each individual donor were used as single-color controls.

Cell growth advantages were calculated using the growth calculation of Eekels et al. for two cell populations, assuming exponential change in the ratio of the two populations over time.^27^ For example, to calculate the growth advantage of GFP-transduced cells over mCherry-transduced cells, we used the formula:

G_a_ = (T_d(GFP)_ – T_d(MCh)_)/T_d(MCh)_ × 100% = – T_d(MCh)_ × log_2_(Y)/(X + T_d(MCh)_ × log_2_(Y)) × 100%

Where G_a_ is the calculated growth advantage, T_d(GFP)_ and T_d(MCh)_ are the doubling time in days of GFP+ and mCherry + cells respectively, X is the number of days of the experiment and Y is equal to % GFP^+^/% mCh^+^ at the time point X divided by the % GFP^+^/% mCh^+^ at the time point 0. T_d(GFP)_ and T_d(MCh)_ were calculated from population doubling curves for each individual donor in these cell culture conditions. The experiment was performed in technical triplicate and for each donor pair we calculated a value for both GFP (pediatric)/mCherry (adult) and mCherry (pediatric)/GFP (adult) crosses.

#### Statistical analysis

Statistical analysis was performed using the R software environment (version 4.0.2) as indicated in the figure legends. Boxplots were generated using the R boxplot function, which displays the first and third quartile as hinges and places whiskers at the most extreme data point that is no more than 1.5 times the length of the box away from the box. Versions for individual packages are listed in the readme file within the GitHub repository for this manuscript (https://github.com/ucl-respiratory/maughan_2022).

## Supplemental information

Document S1. Figures S1–S7

Table S1. Donor characteristics, related to STAR Methods

Table S2. Gene lists used in the study, related to STAR Methods

Table S3. Summary of differentially expressed genes and pathways from transcriptomic experiments, related to Figures 2 and 3

Table S4. RNA sequencing quality control information, related to Figures 2 and 3
